# All-hazards dataset mined from the US National Incident Management System 1999–2020

**DOI:** 10.1038/s41597-023-01955-0

**Published:** 2023-02-24

**Authors:** Lise A. St. Denis, Karen C. Short, Kathryn McConnell, Maxwell C. Cook, Nathan P. Mietkiewicz, Mollie Buckland, Jennifer K. Balch

**Affiliations:** 1 Earth Lab, 4001 Discovery Drive Suite S348–UCB 611, University of Colorado-Boulder, Boulder, Colorado 80309 USA; 2 Cooperative Institute for Research in Environmental Sciences, 216 UCB, University of Colorado-Boulder, Boulder, Colorado 80309 USA; 3 USDA Forest Service, Rocky Mountain Research Station, 5775 US Highway 10 West, Missoula, Montana 59808 USA; 4 Population Studies and Training Center, 68 Waterman Street, Brown University, Providence, Rhode Island 02912 USA; 5 Department of Geography, GUGG 110, 260 UCB, University of Colorado-Boulder, Boulder, Colorado 80309 USA

**Keywords:** Natural hazards, Climate and Earth system modelling

## Abstract

This paper describes a dataset mined from the public archive (1999–2020) of the US National Incident Management System Incident Status Summary (ICS-209) forms (a total of 187,160 reports for 35,170 incidents, including 34,478 wildland fires). This system captures detailed daily/regular information on incident development and response, including social and economic impacts. Most (98.4%) reports are wildland fire-related, with other incident types including hurricane, hazardous materials, flood, tornado, search and rescue, civil unrest, and winter storms. The archive, although publicly available, has been difficult to use for research due to multiple record formats, inconsistent data entry, and no clean pathway from individual reports to high-level incident analysis. Here, we describe the open-source, reproducible methods used to produce a science-grade version of the data, including formal connections made to other published wildland fire data products. Among other applications, this integrated and spatially augmented dataset enables exploration of the daily progression of the most costly, damaging, and deadly environmental-hazard events in recent US history.

## Abstract (summary)

**Table:** 

Measurement(s)	wildfire
Technology Type(s)	NIMS/ICS Reporting
Sample Characteristic - Environment	wildfire
Sample Characteristic - Location	United States

## Background & Summary

There has been a steady rise in the occurrence of billion-dollar environmental disasters in the United States (US) since the 1980s, with the past five years (2017-2021) setting historic highs. The average number of billion-dollar disasters across this period has more than doubled (from 7.1 to 17.8 events per year)^1^. Further, cumulative costs in 2017 set a new annual record of $306.2 billion^2^. There is evidence that the frequency and magnitude of environmental hazards is changing, with some extreme weather events linked to anthropogenic climate change^3^, such as the intensity of tropical storms^4^. Within just the past few decades, the area burned by wildfires in the western US has increased at least threefold^5–8^, with a strong climate change influence in forest systems^9^. This is a critical moment to develop new methods and data products to help understand the interrelationships between the physical and environmental characteristics of environmental hazards, incident response and management actions, and the societal impacts of large-scale or otherwise significant events^10–12^.

Wildfires and hurricanes are two environmental hazards that have significant societal impacts and require costly and complex incident response. In the last five years, damages from wildfires alone have exceeded $81 billion^1^, destroyed over 60k structures^13^, resulted in 233 deaths^1^, and current suppression costs average $2-3 billion each year^14^. Thirteen of the 20 most destructive California wildfires occurred in the past five years (2017-2021), killing 148 people and destroying 40,235 structures^15^. During that same five-year period, there were 18 separate billion-dollar hurricanes that made landfall in the US with an inflation-adjusted total loss of $496.2 billion and 3,474 fatalities^1^. While not all become disasters with great societal impacts, all potentially significant hazard events require a government-coordinated response with critical documentation about how each event is unfolding and its threats to life, property, and other valued resources and assets. In this paper we use the term wildfire to mean fires in wildland fuels caused by unplanned ignitions (natural or human-caused). The broader term wildland fire includes fires from planned ignitions (i.e., controlled or prescribed burns). We use the term hazard to refer to an event or physical/environmental condition with the potential to cause fatalities injuries, property damage, agricultural loss, environmental damage, disruption or daily life, and/or other harm or loss. An incident refers to a specific occurrence of a hazard event requiring emergency response. Disasters are incidents that result in extreme property/resource damages, fatalities, and/or multiple injuries.

The Incident Command System Incident Status Summary Form 209 (ICS-209) captures a unique perspective across an important population of hazard events. It is intended specifically for significant incidents that operate for an extended duration, compete for scarce resources, or require significant mutual aid/additional support and attention^16^. Further, the ICS-209 is intended for use when an incident becomes significant enough to attract media attention, or when there is an increased threat to public safety. The ICS-209 is the authoritative source for in-the-moment observations about the current and forecasted status of an incident, current resources assigned, estimated costs, current and forecasted critical needs, and the societal and natural values currently at threat. These status summaries are required for each operational period of an incident response or when significant events warrant a status update. As a result, these reports offer a unique opportunity to study the relationship between hazard characteristics, incident response, and the societal impacts/threats incrementally across all phases of active response. The ICS-209 was originally developed to manage large or otherwise significant (high complexity) wildfires and later adapted for all-hazards use^17^. Much of the information captured on the form is specific to wildfire, and over 98% of the incidents in the dataset are wildfires. A wildfire is generally considered large enough to require an ICS-209 report when it exceeds 100 acres in timber or 300 acres in grass or brush. Although only 1–2% of wildfires become large (e.g., >300 acres), those incidents account for approximately 85% of total suppression costs and upwards of 95% of total acres burned each year^18^.

Annually archived ICS-209 data have been used in research^19–27^ relating wildfire activity to weather, fuel treatment activities, firefighting response, and a range of socioeconomic impacts. However useful, the data were not originally collected with research applications in mind, and the raw data must be carefully prepared for scientific analyses. We completed an initial effort to produce a research-ready, science-grade compilation of the archived data, published as the ICS-209-PLUS data product^28^. That work included standardising and merging data collected using different versions of the input form and electronic archive, cleaning and aligning with the current system, smoothing values across reports, and facilitating incident-level analysis with formal connections to other published wildland fire datasets. The initial product has since been used in several research projects^29–31^. This paper describes the methods used to expand and improve upon the original data product, including the addition of six years’ worth of data so that the ICS-209-PLUS dataset now spans 1999-2020. We also describe new components of the dataset that further facilitate analyses related to the burned area time series for wildfire incidents and to the spatiotemporal array of wildfire impacts. We characterize high-level spatial and temporal wildfire distributions across several key variables. We then compare these values with a larger population of wildfires in the US. We then look in detail at relationships between these variables for a major wildfire event, the 2017 Chetco Bar Fire, examining linkages to satellite-derived datasets. We conclude by identifying key opportunities for use of this dataset and describing how this open-source solution could be extended in the future.

## Methods

### Primary data source

Historical data from the ICS-209s are now archived by the National Wildfire Coordinating Group (NWCG) on the Fire and Aviation Management (FAM) Application and Information Website at (https://famit.nwcg.gov/applications/SIT209/historicalSITdata), which replaces the previous Fire and Aviation Management website (FAMWEB) homepage and security portal. All data in the ICS-209-PLUS dataset are sourced from the raw data files, and the dataset is designed so that it can be extended as new data are released. We manually downloaded the raw data from the FAM archive available at the time of processing, saving each table in Excel format. The data span three separate versions that we will refer to as Historical System 1 (HIST1) from 1999 to 2002, Historical System 2 (HIST2) from 2001 to 2013, and the current version (CURRENT) from 2014 to the current annual archive. The input tables for each of these versions are summarised in Table 1 below. The ICS-209-PLUS dataset was initially produced through 2014 due to missing source tables and data duplication issues in 2015 to 2017. Additional issues in the current system needed to be addressed prior to publication. These fixes are described in the *Record Repair Methods 2014* + section below.

**Table 1:** Input Table Names.

Table Content	System Version	Table Name(s)
Fire Complex Record	Historical System 2*	IMSR_IMSR_209_INCIDENT_COMPLEX
	Current System	SIT209_HISTORY_INCIDENT_COMPLEX_ASSOCS
Incident Record	Current System	SIT209_HISTORY_INCIDENTS
Daily Situation Report	Historical System 1	IMSR_INCIDENT_INFORMATIONS
	Historical System 2	IMSR_IMSR_209_INCIDENTS
	Current System	SIT209_HISTORY_INCIDENT_209_REPORTS
Current Resources	Historical System 1	IMSR_INCIDENT_RESOURCES
	Historical System 2	IMSR_IMSR_209_RESOURCES
	Current System	SIT209_HISTORY_INCIDENT_209_RES_UTILIZATIONS
Structural Information	Historical System 1	IMSR_INCCIDENT_STRUCTURES
	Historical System 2	IMSR_IMSR_209_INCIDENT_STRUCTURES
	Current System	SIT209_HISTORY_INCIDENT_209_AFFECTED_STRUCTS
Casualties & Illnesses	Current System	SIT209_HISTORY_INCIDENT_209_CSLTY_ILLNESSES
Life Safety Management	Current System	SIT209_HISTORY_INCIDENT_209_LIFE_SAFETY_MGMTS
Suppression Strategies	Current System	SIT209_HISTORY_INCIDENT_209_STRATEGIES
Lookup Tables	Historical System 1, Historical System 2	IMSR_LOOKUPS, IMSR_STATES
	Current System	SIT209_HISTORY_SIT209_LOOKUP_CODES, COMMONDTA_NWCG_UNITS, COMMONDATA_STATES

*2010 to 2013 only.

### History of ICS

The ICS-209 form, commonly referred to as a *sitrep*, is part of the US National Incident Management System/Incident Command System (NIMS/ICS). The earliest implementation of ICS was developed by the US Forest Service (USFS) following a devastating fire in California in 1970 that claimed 16 lives, destroyed over 700 structures and burned over 500k acres. Numerous communication and coordination issues hampered the effectiveness of the agencies involved, resulting in a congressional mandate requiring the USFS to design a new system to facilitate interagency coordination and to support the allocation of suppression resources in dynamic, multi-fire situations^17,32,33^. The USFS worked in collaboration with California state agencies to produce FIRESCOPE (FIrefighting RESources of California Organized for Potential Emergencies) with two key components: the Incident Command System (ICS) and the Multi-Agency Coordination System (MACS)^32^. By 1981, FIRESCOPE was used by agencies throughout Southern California and was adapted for non-fire use. In parallel with this effort, the NWCG adopted and revised the FIRESCOPE ICS documentation to create the National Interagency Incident Management System (NIIMS) Incident Command System Operational System Description (ICS 120-1) - a document that was collectively maintained by CalFire and NWCG. This document later served as the basis for NIMS ICS^33^. Following the 2001 September 11th terrorist attacks in the US, the Department of Homeland Security (DHS) was formed, and on February 28, 2003, President George W. Bush issued Presidential Directive-5^34^ calling for the establishment of a single, comprehensive national incident management system, which became NIMS.

### NIMS ICS

The NIMS was issued in March 2004 to enable responders at all jurisdictional levels and disciplines to work together more effectively by establishing a single, comprehensive national incident management structure^17,33^. In 2005, there was a push to institutionalize the use of ICS across the entire response system and by 2006, federal funding for state, local and tribal grants was tied directly to compliance with the NIMS^33^. The NIMS/ICS is built upon existing incident management best practices including ICS and MACS. It fully delineates standardized command and control structures and procedures designed to support interoperability among jurisdictions and across disciplines as the complexity of a response effort increases. The planning function is centralized within ICS with information captured during each operational period flowing up to the tactical and strategic planning level^17^.

### The ICS 209 incident status summary

The Federal Emergency Management Agency (FEMA) describes the purpose of the ICS-209 as follows: “*The ICS 209 is used for reporting information on significant incidents … The ICS 209 contains basic information elements to support decision making at all levels above the incident to support the incident. Decision makers may include the agency having jurisdiction, but also all multiagency coordination system (MACS) elements and parties, such as cooperating and assisting agencies/organizations, dispatch centers, emergency operation centers, administrators, elected officials, and local, tribal, county, State, and Federal agencies.”*^16^. The ICS-209 is described as providing a “snapshot in time,” capturing the most accurate and up-to-date information available at the time of preparation. The form is typically completed by the Situation Unit Leader or Section Planning Chief within the Incident Management Team but may also be completed by a local dispatcher or another staff member when necessary. Reports are logged for each operational period or when information becomes outdated in a quickly evolving incident. Each report describes current characteristics of the hazard, current environmental conditions, current and projected incident management costs, details about specific resources assigned to the incident, critical resource needs, a description of structural and life safety threats, an ongoing accounting of injuries, fatalities, damages, and the projected incident management outlook.

The format and content of the ICS-209 has evolved over time in parallel with efforts to adapt the form for all-hazards use. Our inspection of records identified new fields added in 2004, when the system was incorporated into NIMS, and again in 2007 to support all-hazards reporting. It is important to note that the use of NIMS/ICS was not mandatory on large incidents until fiscal year 2006^33^, and so there may be significant gaps in reporting prior to this date. Any time-series analysis exploring trends in the data must acknowledge this limitation. Additionally, the ICS-209-PLUS dataset is based on the published data and may exclude records containing sensitive information.

The raw data are published in three separate formats. The original format, Historical System 1 (HIST1), spans 1999 to 2002 and includes basic incident information, start location, personnel usage, and total structures damaged/destroyed. The second format, Historical System 2 (HIST 2), was introduced in 2001 and captures a broader set of information related to the hazard including incident complexity, fire behaviour, fuels, and local weather. It contains freeform narrative text fields to capture projected risk to communities, resources at risk, critical resource needs, planned actions, and projected hazard movement/spread. Additional societal impact values include injuries, fatalities, evacuations in progress, and estimates of structures threatened. The current system format was released in 2014. Tighter standardisation of values on the form resulted in cleaner categorical data. Major changes include an expansion of formats for capturing point-of-origin data, expanded functionality for tracking casualties and illnesses, and expanded functionality for tracking life safety management. Table 2 provides a high-level summary of data elements in the ICS-209-PLUS dataset. Refer to^35^ for a description of individual fields in the ICS-209-PLUS *sitrep* table. Additionally, the FEMA ICS-209 Form^16^ describes the intended use of each field in the ICS-209 form and whether the field is required.

**Table 2:** Description of Data Elements by Type on ICS-209 Incident Status Form.

Information Type	Data Elements
Incident Reporting Detail	Incident Name, Incident Number, Reporting Time Period, Report Status (Initial, Update, Final), Approval and Routing Information
Current Incident Status	Incident Commander(s), Incident Management Organization, Level of Complexity, Percent Contained/Completed, Estimated Costs to Date, Current Resources (Personnel and Equipment Currently in Use), Agencies Involved, Additional Cooperating and Assisting Agencies, Fire Complex Details
Hazard Description & Conditions	Incident Type, Cause, Start Date/Time, Location, Current Area Involved, Materials/Hazards Involved, Fuels, Fuel Conditions, Fire Behavior, Current & Forecast Weather
Projected Outlook & Needs	Critical Resource Needs, Current and Projected Weather/Conditions, Projected Activity/Movement/Escalation or Spread, Strategic Objectives, Planned Actions, Projected Final Size, Projected Final Costs, Projected Containment Date, Projected Demobilization Date
Societal Impacts	Structures Threatened/Damaged/Destroyed, Values at Risk, Injuries, Fatalities

### The ICS-209 situation report & related tables

The situation report or *sitrep* table contains the majority of fields in the *Incident Status Summary*. In addition to this, all three versions have a *resources* table that tracks personnel and equipment by response agency. The earliest system (Historical System 1) tracks the fewest number of resource types (*Crew 1*-3, *Helicopter 1*-*3* and *Overhead Personnel*) with estimated *Total Personnel* stored directly in the *sitrep* table. Later versions expand on these resource types and add a text field to the *sitrep* table to capture additional cooperating agencies. We pivot the *resources* tables by agency to calculate *Total Personnel* for each *sitrep*. All three versions also have a *structures* table that tracks the number of structures threatened, damaged, or destroyed by commercial, residential, and outbuilding structure types. We pivot the structures table by structure type to calculate totals related to structures threatened, damaged, or destroyed for each *sitrep*. Both historical versions track injuries and fatalities directly in the *sitrep* table, whereas the current system tracks this data in the *Casualties and Illnesses* table. This new table introduces a broader range of individual impacts including the number of people missing, trapped, evacuated, sheltering in place, in temporary shelters, immunized, and quarantined. Additionally, the *Life Safety Managements* table in the current system keeps track of incident management activity related to mass notifications, area restrictions, evacuations, immunizations, quarantine and sheltering-in-place. We use the *Casualties and Illnesses* table to calculate the number of injuries and fatalities and evacuation status for each *sitrep*. This could be expanded in future releases to track a broader range of life safety threat and current response status. Finally, there is an annual *Lookup Codes* table containing standardised field values. These values have remained fairly consistent with new values added over time. Current code values are summarized in the standard codes reference^16^. Any modifications to existing values are discussed in the *Transforming Standardised Fields* section below.

There are two parent tables related to the *sitrep* table: an *incidents* table and a *complexes* table. The complexes table was added in 2010. A complex is defined as *two or more individual incidents located in the same general area which are assigned to a single incident commander or unified command*^33^. The complex record clusters all fires and *sitreps* associated with a fire complex under the same incident number, capturing individual fire names, suppression strategies, current containment percentages and estimated costs to date. The current version also includes the current area for individual fires within the complex. This information is missing in both historical versions of the system. We used data that was manually compiled and verified to derive the *Wildfire Complex Associations* table for wildfires between 1999 and 2010 and data derived initially from the *complex associations table for* 2010+ to describe the relationship between fire complexes and individual fires. This process is detailed in the methods below. Finally, the current version has an incidents table that contains basic incident level information including discovery date, cause, area, location, and estimated cost to date. Concatenated versions of the original complex and incident tables are included in the dataset (Table 1), but we did not clean or modify these tables. We created a new *Wildfire Incident Summary Table*^35^ derived from the cleaned and smoothed values in the Wildfire *sitreps* table. This new incident level table contains additional incident level statistics that enhance the research value of the dataset. This new table is described in detail in the methods section.

### Open/Reproducible framework

We produced the ICS-209-PLUS dataset using principles of open and reproducible science^36–38^. All data source files, and the final ICS-209-PLUS dataset are archived online^35^. The python source code for the ICS-209-PLUS creation^39^, R source code to link to the Fire Events Delineation (FIRED) dataset^40^ and the spatiotemporal linkage and all figures and tables^41^, are publicly available. Our aims are twofold: to provide transparency to the methods and assumptions used to produce the final dataset and to provide a framework for others to adapt or expand upon the dataset. The code is written in Python using the Numpy and Pandas data science libraries. We were unable to automate the downloading of the raw data from FAM archive and so our code assumes all relevant tables (Table 1) are downloaded to the corresponding annual directories beforehand.

We accomplished several key objectives in this updated version. First, we aligned data elements and standardised values across both historical versions with the current data model, extending the record through 2020. This allows for seamless comparison of records across the entire time period. Secondly, due to the free-form nature of the fields and limited mechanisms enforcing data entry standards, the original data is notoriously messy and difficult to use. The scripts are designed to automate as much of the cleaning and formatting as possible, improving the overall consistency of the dataset. It is also designed to support the manual updates identified in the process of producing the dataset. This is important for several reasons. It allowed us to easily incorporate updates for fields such as *Latitude* and *Longitude* that were deemed critical for dataset use. In the initial release it provided a framework for incorporating the cleaning efforts and refinements curated by co-author Karen Short and for repairing issues identified in the *Current* version found in years 2015+ (see *Repairing Records 2015+*). New fields have been added based on values available in the current reporting system (see *Extending the Dataset Beyond 2014*). We connect the ICS-209-PLUS dataset with the Fire Program Analysis Fire-Occurrence Database (FPA FOD^42,43^) enabling linkage with Monitoring Trends in Burn Severity (MTBS^44,45^) fire products and additionally connect to the novel spatial fire database, Fire Events Delineation (FIRED^46,47^) (see *Linking the Wildfire Incident Summary Record to FIRED)*. The updated dataset is then used to create a spatiotemporal linkage, which assigns wildfire incidents to corresponding county, tract, and block group units as well as quarters and years (see *Assigning Wildfire Incident IDs to Spatial and Temporal Units*). The following sections detail how the dataset is produced from the merging of the original source files to the creation of the *Wildfire Incident Summary* table, and external linkages.

### Producing the ICS-209-PLUS dataset

**Fig. 1:**
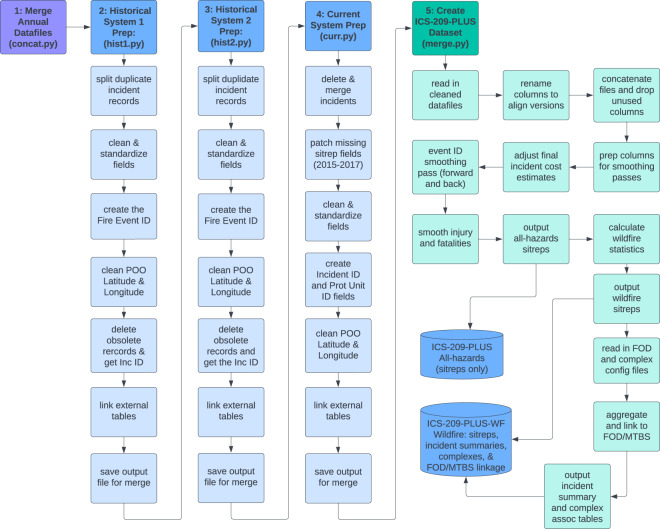
Process Followed to Create the ICS-209-PLUS All-hazards and Wildfire Datasets.

The ICS-209-PLUS dataset is produced by a series of Python scripts that first consolidates the annual files for each of the tables across the three versions of the system (Table 1). Each version of the *sitrep* table is then cleaned and prepared for the merge. This includes general cleaning and formatting for each field, field-level updates to correct known errors, and deletion of duplicates/erroneous records. Each of the related tables are pivoted and totals are calculated for personnel, aerial equipment, structures threatened, structures damaged, structures destroyed, injuries, fatalities, evacuations (2014+), and wildfire suppression strategy (2014+) for each situation report. These totals are joined into the situation report and then columns across the three versions are aligned and appended together. Once the data has been consolidated into a single dataset, individual fields are cleaned and smoothed, filling missing values and adjusting values where appropriate. This finalised version is then used to produce an all-hazards dataset (ICS-209-PLUS All-Hazards), and a wildfire dataset (ICS-209-PLUS WF). The wildfire dataset is composed of two tables: all the wildfire daily status summaries and an incident level summary record. The *Wildfire Incident Summary* contains high-level statistics that are useful from a research standpoint. An overview of the process followed to create both the all-hazards and wildfires datasets is summarized in the flowchart below (see Fig. 1). The python code containing the logic for each step is included in parenthesis.

### Cleaning and formatting the individual datasets

The scripts clean each version of the *sitrep* table prior to the merge. This is necessary to deal with subtle differences between each version. Unique identifiers are constructed within the historical datasets to separate out individual fire events and to group related incidents together. We clean and standardise values for each historical version so that they merge smoothly into the final dataset. Once this preliminary cleaning is complete, members of the historical dataset are compared with a refined version of the record and *sitreps* that are not members of this refined set are archived to a *deleted sitreps* table (described later).

### Creating unique incident and fire identifiers

The *Incident Number* field is meant to uniquely identify an incident, but there are multiple issues with this field, particularly in the historical datasets. In some instances, incident numbers are incomplete, or they are re-used from year to year, resulting in *sitreps* for multiple incidents being grouped together as a single incident. Splitting them based on year is problematic because some fires, particularly in the southeastern United States span the annual boundary or have a final report filed in the next year. There are also instances where Incident Name and point of origin are distinctly different but share the same incident number. Conversely, there are incidents in the current version that have the same incident number, but are split across multiple unique system identifiers. Finally, there are instances where fires are incorporated into a fire complex and the *Incident Number* changes to that of the fire complex. We addressed these issues by creating two concatenated ID fields: the *Fire Event ID* and the *Incident ID*. The *Fire Event ID* is used to identify individual wildfires regardless of whether they are managed as part of a larger fire complex. The *Incident Id* is used to group all sitreps related to an incident response, clustering related situation reports that are related but may differ in terms of the *Incident Number* and or the *Incident Name*.

### The fire event ID

The *Fire Event ID* is a concatenation of the *Start Year* and the *Incident Number* fields followed by a sequence number (default = 1). The *Start Year* separates instances where the *Incident Number* is re-used from year-to-year. We manually scanned sitreps in both historical versions sorted by *Incident Number, Incident Name, Discovery Date*, and the report date to identify records that needed to be split. For example, Incident Number “AR-ARS-D2” was assigned to three separate incidents starting in different locations at different times (Table 3). We split them by adjusting the sequential variable for Dierks to 2 and Red Barn to 3.

**Table 3:** Example of Fire Event ID Splitting Three Separate Wildfires Sharing Same Incident Number.

Incident Name	Location	Coordinates	Discovery Date	Fire Event ID
Vandervoort	3 miles NE of Vandervoort AR	33.134167, −93.858333	2011-04-03 21:41:00	2011|AR-ARS-D2|1
Dierks	7 miles NE of Dierks	34.145833, −93.895	2011-04-03 15:12:00	2011|AR-ARS-D2|2
Red Barn	East of Cowlingsville	33.869167, −94.087778	2011-09-10 15:30:00	2011|AR-ARS-D2|3

### The incident ID

The *Incident ID* is a concatenation of the *Start Year*, the final *Incident Number*, and the final *Incident Name*, such that multiple fires can be grouped together if they are later incorporated into a larger response. This information is missing in the historical datasets, but was manually compiled and verified by co-author Karen Short over time during the compilation of the Fire Program Analysis Fire-Occurrence Database (FPA-FOD^42,43^). That work included consulting several sources to piece together relationships between individual fires and fire complexes and to purge duplicate and erroneous situation reports across the two historical records. We use this cleaned version of the ICS-209 records, referred to as the Short master list^35^, as a definitive reference such that this table is used to create the *Incident ID* for all historical sitreps and determines which records should be deleted (see *Purging Duplicate and Erroneous Records*).

The example below is taken from the 2006 Boundary Complex. It illustrates how *Incident ID* is used to group related sitreps together (Table 4) while preserving the original identifiers. The complex includes the following individual fires: Boundary, Elkhorn 2, Lost Lake, Deer, Thicket, Chuck, East Elk, North Elk, and Knapp 2, all under *Incident ID* 2006_ID-SCF-006336_BOUNDARY COMPLEX, which also has its own sitreps. The *Fire Event IDs* are included at the far right to illustrate how the *Incident ID* allows for multiple physical fires to be grouped together as a single response, whereas the *Fire Event ID* provides a unique identifier for each physical fire event or management grouping.

**Table 4:** Multiple Fires Grouped Within 2006_ID_SCF-006336_BOUNDARY COMPLEX.

Incident Number	Incident Name	# sitreps	Start	Fire Event ID
ID-SCF-006336	Boundary	8	8/21	2006|ID-SCF-006336|1
ID-SCF-006336	Boundary Complex	39	8/8	2006|ID-SCF-006336|1
ID-SCF-6245	Elkhorn2	1	8/9	2006|ID-SCF-6245|1
ID-SCF-6349	Lost Lake	2	8/8	2006|ID-SCF-6349|1
ID-SCF-6369	Deer	2	8/31	2006|ID-SCF-6369|1
ID-SCF-6373	Thicket	1	8/7	2006|ID-SCF-6373|1
ID-SCF-6415	Chuck	3	8/9	2006|ID-SCF-6215|1
ID-SCF-6494	East Elk	1	8/21	2006|ID-SCF-6494|1
ID-SCF-6496	North Elk	2	8/21	2006|ID-SCF-6496|1
ID-SCF-6554	Knapp #2	1	9/7	2006|ID-SCF-6554|1
ID-SCF-6554	Knapp 2	4	9/7	2006|ID-SCF-6554|1

### General field level cleaning

We used the Python data science tools to inspect values contained in each column across the three versions to determine what actions were needed to clean and prepare for the merge. Many columns had standardised values, but contained extraneous characters or inconsistencies. The script uses regular expressions to standardise values for fields like *GACC Priority, Dispatch Priority, Percent Containment, Containment Date*, and *Incident Management Team Type* fields. Once these values are standardised, they are linked to corresponding values in the lookup code tables. The script also removes all linefeeds and hidden characters from text fields to make viewing and processing the fields easier. Values such as “N/A”, “same”, or “none” and redundant values are deleted from the consolidated text fields. The script fixes any obvious date errors (e.g., year values of 1901 instead of 2001) and applies consistent formatting across all date fields. All *Latitude* and *Longitude* values have been converted to decimal degrees. We cleaned and formatted most of the fields except weather variables and fuels. We determined that both these fields would require extensive effort and fell outside the scope of the current release.

Throughout the process, we identified individual values that were clearly an error and made some individual field level updates. These updates are limited and are incorporated into the general field cleaning function for each script. In the future, there is potential to maintain these updates as part of a field level update table that could be loaded at runtime to automate individual field-level modifications. This would be an ideal solution to support ongoing update and maintenance of the dataset in the future but is beyond the scope of the current release.

### Transforming standardised fields

Standard values remained relatively consistent across the three versions, with new values added as the form was adapted for all-hazards use. The *Cause* and *Suppression Method Abbreviation* fields changed slightly from the historical to the new version and so we translated old values to equivalent new values (Table 5). A handful of Incident Types were eliminated in the current system. After careful consideration, we decided to keep the historical values for consistency and to prevent information loss. Prescribed burns (RX) and Wildfire Used for Resource Benefit (WFU) have been included in the Wildfire datasets. The ICS-209-PLUS Form is not intended for tracking planned ignitions and so the RX incident type is rare (0.4% of incidents), but the form was sometimes used to request resources during periods of resource scarcity. There are only 144 incidents (471 sitreps) with Incident Type RX from 1999 to 2013 with most occurring in 2005 (31 incidents). The WFU Incident Type was obsoleted in 2009. There are 772 Incident Summary records and 6120 sitreps for that type prior to 2009. We reclassified all values that were binary (yes/no) to boolean values (true/false) to make them consistent and to put them in a more standard database format.

**Table 5:** Standard Field Values Eliminated or Updated to New Values.

Field	Version	Original Value	New Value
Cause	Hist1, Hist2	**N** (No Description)	**O** (Other)
Complex	Hist2 Current	**Y/N** **S/C**	**True/False**
Evacuation In Progress	Hist2, Current	**Y/N**	**True/False**
Incident Type Abbreviation	Hist1, Hist2	**SAR** (Search & Rescue) **USR** (Urban Search & Rescue)	**SR/R**
		**BAR** (Burned Area Emergency Rehabilitation) **LE** (Law Enforcement) **MC** (Mass Casualty) **OS (**Oil Spill) **RX** (Prescribed Burn) **STR** (Structure Fire) **WFU** (Wildfire for Res Benefit)	**No Change –** Values Preserved to Prevent Data Loss
Suppression Method Abbreviation	Hist2	**CC** (Confine) **MM** (Monitor) **PZ** (Point Zone Protection)	**C** (Confine) **M** (Monitor) **PZP** (Point Zone Protection)

### Cleaning and consolidating narrative text

Each version of the *Incident Status Summary* provides space for recording important observations from incident command. The earliest version of the report (Historical System 1) has only one *Narrative* field whereas later versions have multiple narrative text fields organized around the following topics: critical resource needs, current threats, projected incident movement and spread, weather, fuels, relevant conditions, and general remarks. Critical resource needs, current threats, and projected fire activity capture projected values at 12, 24, 48, 72, and greater than 72 hours from the current report. We consolidated these observations into one narrative field for each topic to manage the complexity of the dataset, eliminate redundancy, and to organize the observations for potential text mining and topic modeling efforts. Before consolidating, we clean each individual field to strip hidden characters, eliminate placeholder values (e.g. “n/a”, “same”, “none”) and eliminate duplicate values. A pipe ‘|’ character is used to separate observations. For example, the following entries in the projected activity fields:

Projected Movement 12: *“Minimal fire movement due to lower temps higher RH and precipitation.”*

Projected Movement 24: *“Minimal fire movement due to lower temps higher RH and precipitation.”*

Projected Movement 48: *“Moderate fire activity is anticipated on Friday due to warming temps, falling RH, and wind.”*

Projected Movement 72: *“same”*

are consolidated into a single *Projected Activity Narrative*:

*“Minimal fire movement due to lower temps higher RH and precipitation|Moderate fire activity is anticipated on Friday due to warming temps, falling RH, and wind.”*

Table 6 summarizes the narrative text fields in the final dataset. The boldfaced fields are the newly consolidated fields that condense projected values into a single narrative summary. The version column identifies which versions populate this field.

**Table 6:** Narrative Fields in Final Dataset.

Field Name	Description	Version
ADDTNL_COOP_ASSIST_ORG_NARR	List of Additional Agencies Not Tracked in Resources Table Cooperating on Fire.	HIST2, Current*
**CRIT_RES_NEEDS_NARR**	Projected Resource Needs at 12, 24, 48, 72, greater than 72 hours	HIST2, Current
**CURRENT_THREAT_NARR**	Current Resources and Values at Risk at 12, 24, 48, 72, greater than 72 hours	HIST2, Current
HAZARDS_MATLS_INVOLVEMENT_NARR	Description of Fuels and Materials Involved in Fire.	HIST2, Current
LIFE_SAFETY_HEALTH_STATUS_NARR	Summary of Current Risk to Life and Health Safety.	Current Only
MAJOR_PROBLEMS	Summary of Any Major Problems	HIST2 Only
OBS_FIRE_BEHAVE	Description of Fire Behavior	HIST2 Only
PLANNED_ACTIONS	Summary of Planned Actions	HIST2, Current
**PROJECTED_ACTIVITY_NARR**	Projected Hazard Activity at 12, 24, 48, 72, greater than 72 hours	HIST2, Current
REMARKS	General Remarks Field	HIST1, HIST2, Current
SIGNIF_EVENTS_SUMMARY	Summary of Significant Events for the Current Operational Period	HIST2, Current
STRATEGIC_NARR	Strategic Objectives and Strategic Discussion Fields (Current System Only)	Current Only
**WEATHER_CONCERNS_NARR**	Current and Projected Weather Outlook, Consolidates Weather Observations from HIST2 Combined into Single Field.	HIST2, Current

#### Extending the dataset beyond 2014

We incorporated new values from the *Current System* daily situation reports when we extended the dataset beyond 2014. This includes new descriptors for fire behavior, point of origin, trail and road closure flags, estimates of current evacuations, and protection unit information. The *Current System* breaks down injuries and fatalities by emergency personnel or the general public. The Suppression Method also allows for multiple suppression methods and percentage reporting across corresponding strategies. Table 7 summarizes these new fields with the *Table* column indicating if the field is in the *sitreps* table or the *Wildfire Incident Summary* table.

**Table 7:** New Situation Report and Incident Summary Fields.

Field Name(s)	Description	Data Type	Table
AREA_CLOSURE_FLAG	Area Closure Flag	True/False	Sitrep
ANTICIPATED_COMPLETION_DATE	Expected Completion of Planned Response	Date/Time	Sitrep
FATALITIES_PUBLIC	Number of Public and Emergency Responder Fatalities	Numeric	Incident
FATALITIES_RESPONDER			
FIRE_BEHAVIOR_1	Primary Secondary, and Tertiary Fire Behavior Descriptors, (e.g. Torching, Creeping, Flanking)	Categorical	Sitrep
FIRE_BEHAVIOR_2			
FIRE_BEHAVIOR_3			
GEN_FIRE_BEHAVIOR	General Fire Behavior Descriptor: Minimal, Moderate, Active, or Extreme	Categorical	Sitrep
IRWIN_ID	Unique Identifier from the Integrated Reporting of Wildland-Fire Information (IRWIN) Service	Text	Sitrep
NWCG_IDENTIFIER	Unique Identifier from the National Wildfire Coordinating Group Common Data NWCG Units Table	Numeric	Sitrep
PEAK_EVACUATIONS	Peak Evacuations Reported	Numeric	Incident
PERCENT_C	Percent Supression Method Split Across each Categorical Value: Confine (C), Full Suppression (FS),Monitor (M) and Point Zone Protection (PZP)	0–100%	Sitrep
PERCENT_FS PERCENT_M			
PERCENT_PZP			
POO_US_NGR_XCOORD	Point or Origin Specified in the US National Grid Reference System	Mixed Numeric and Alpha-Numeric	Sitrep
POO_US_NGR_YCOORD			
POO_US_NGR_ZONE			
POO_UTM_EASTING	Point of Origin Specified in the Universal Traversal Mercator (UTM) Coordinate System	Numeric	Sitrep
POO_UTM_WESTING			
POO_UTM_ZONE			
PROT_UNIT_ID	Unique Identifier for Agency with Protection Responsibility based on the Point of Origin	Numeric	Sitrep
PROT_UNIT_NAME	Name of Agency with Protection Responsibility	Text	Sitrep
PROT_UNIT_TYPE	Protection Unit Type (e.g. State, Federal, Tribe, County & Local)	Categorical	Sitrep
ROAD_CLOSURE_FLAG	Road Closure Flag	True/False	Sitrep
RPT_EVACUATIONS	Estimate of Current Evacuations	Numeric	Sitrep
RPT_P_FATALITIES RPT_R_FATALITIES	Number of Public and Emergency Responder Fatalities for This Report	Numeric	Sitrep
RPT_P_INJURIES RPT_R_INJURIES	Number of Public and Emergency Responder Injuries for This Report	Numeric	Sitrep
SUP_METHOD_FINAL	Final Suppression Method Reported	Categorical	Incident
SUP_METHOD_INITIAL	Initial Suppression Method Reported	Categorical	Incident
SUP_PERCENT_FS	Percent of Full Suppression Reported	0–100%	Incident
SUP_SERIES	List of Suppression Methods by REPORT_TO_DATE (e.g. [PZP,FS,FS])	List	Incident
TRAIL_CLOSURE_FLAG	Trail Closure Flag	True/False	Sitrep

For brevity related fields are listed here together but are separate data elements in the data product.

### Point of origin coordinates

Given the critical role point of origin data plays in geospatial analysis, we manually cleaned and inspected the point of origin coordinates, fixing obvious errors and providing estimates for missing or obviously erroneous values. The values in the earliest system (Historical System 1) were first converted from degrees and minutes to decimal format. We then mapped all the points, identifying those that fell outside of the United States and its territories. The most common issue was an incorrect numeric sign for latitude or longitude. The longitude was incorrect for 98.5% of the reported locations in the second historical system (Historical System 2). Nearly all values in the Current System (99.72%) fell within the United States and its territories. Wherever possible, we used latitude/longitude values from the FPA FOD for missing and erroneous values. We then manually examined the remaining values that fell outside of the clipped boundaries individually. We used the information contained in other point of origin fields (e.g., the location description) to estimate latitude and longitude. For each of these estimated values, we set the *Lat/Long Update* flag to true and set the *Lat/Long Confidence* field to capture our level of confidence in this estimate (low, medium, high). We rated our estimate as high to medium if we were able to get close to the actual point of origin (e.g., intersection of roads) and low if the location description was vague (e.g., 6 miles southwest of Sisters, Oregon). Our goal was to maximize available geospatial information while allowing users of the data to filter out low-confidence or updated values when a high level of accuracy is needed. The accuracy and completeness of the data improves over time across the three versions, as well as the location description fields available for estimation. In the earliest version (Historical System 1), 29% of coordinates were missing or erroneous but we were able to populate nearly half (49%) of the missing values with estimates taken from the corresponding record in the FPA-FOD database. With limited information, we were only able to derive a point of origin for 103 additional values (12%) with the majority of those (89 of the incidents) estimated as low confidence due to limited location information.

In contrast, only 2% of the coordinates were missing or erroneous in the second historical version (Historical System 2) and we were able to populate 45% of the missing values with estimates taken from the corresponding record in the FPA FOD. We were able to estimate an additional 26% of missing values with a mix of confidence levels (42 incidents with high confidence, 26 with medium confidence, and 50 with low confidence levels). Finally, only 1.6% of the coordinates were missing or erroneous in the 2014+ data with over half of the missing values populated with values taken from the corresponding record in the FPA FOD, and we were able to estimate all but 2 of the remaining values with a high level of confidence with roughly half requiring a simple swap of latitude and longitude values to correct. Table 8 below summarizes latitude and longitude updates by system and corresponding levels of confidence.

**Table 8:** Latitude/Longitude Updates by System Version.

Version	High confidence	Medium Confidence	Low Confidence	No Value
Historical 1	447 (438 FOD)	5	89	346
Historical 2	238 (201 FOD)	26	50	130
Current	31 (23 FOD)	0	0	2
Total	716	31	139	478

Some of these records may get deleted as part of the merging and cleaning process described below resulting in 98% of incidents with a valid point of origin in the final dataset.

### Preparing to merge

The individual fields and values in the incident status reports remained relatively consistent across the three versions, but the underlying data model continued to evolve to adapt to all-hazards management and to capture more detailed information about resources, life safety threats, and management actions. Our goal when mapping values across the three versions was to maximize continuity while making the historical data forward compatible with the current system. Most of the data elements aligned with minimal or no modification. There were several data elements that had no equivalent in the current system. We preserved the ones that had a high fill rate: *Major Problems, Observed Fire Behavior*, and *Terrain*. A list of the resulting columns, including their fill rates, is provided in the ICS-209-PLUS Sitrep Table Definition file^35^.

In addition to the consolidated text fields described in the section above, we added fields to the situation report to align the historical data with the current version or where they added value. For example, the *Acres* field provides a convenient way to compare incident area without having to convert units of measurement. The day of year (DOY) fields (*Discovery DOY and Report DOY)*, *Start Year*, and *Current Year* (CY) support simple querying and analysis without having to manipulate the related timestamps. The script also integrates totals calculated from the related tables into the incident status record. Table 9 summarizes the new values that have been added to the *Incident Status Summary* table.

**Table 9:** Fields added to the Incident Status Summary Reports (sitrep) table.

Field Name	Description
ACRES	Current size in acres
COMPLEX	True/False indicating incident is part of a fire complex
COMPLEX_NAME	Fire complex name (may or may not be same as *Incident Name*)
CRITICAL_RES_NEEDS_NARR	Critical resources identified for upcoming 12/ 24/ 48/ 72/72 + hours
CURRENT_THREAT_NARR	Current values at risk for upcoming 12/24/ 48/ 72/ 72 + hours
DISCOVERY_DOY	Julian day of the current year for the *Discovery Date* field
EVACUATION_IN_PROGRESS	True/False evacuations in progress
FATALITIES	Current number of reported fatalities
FIRE_EVENT_ID	Unique Identifier for individual fire events
INCIDENT_ID	Unique Identifier for all sitreps grouped under the same incident response
INJURIES	Number of injuries for this reporting period
INJURIES_TO_DATE	Number of injuries to date
NEW_ACRES	Number of acres since the last report
REPORT_DOY	Julian day of the current report
STARTYEAR	Start year of the incident
STR_DAMAGED_RES	Total residential structures damaged, destroyed, threatened for the current operational period.
STR_DESTROYED_RES	
STR_THREATENED_RES	
STR_DAMAGED	Total structures damaged, destroyed, threatened for the current operational period.
STR_DESTROYED	
STR_THREATENED	
STR_DAMAGED_COMM	Total commercial structures damaged, destroyed, threatened for the current operational period.
STR_DESTROYED_COMM	
STR_THREATENED_COMM	
STR_DAMAGED_RES	Total residential structures damaged, destroyed, threatened for the current operational period.
STR_DESTROYED_RES	
STR_THREATENED_RES	
TOTAL_AERIAL	Total number of aerial support resources currently assigned to the fire.
TOTAL_PERSONNEL	Total number of personnel resources summed across all agencies

### Purging duplicate and erroneous records

As mentioned above, the historical datasets overlap between 2001 and 2003, and sometimes incident status reports were logged in both systems resulting in duplicate records across the two systems, along with other erroneous records. Many of these records were deleted from the dataset originally maintained by Karen Short. Rather than deleting each of these records explicitly, we use the records in the Short dataset as a master list.

Any wildfire that does not exist in the master list is removed from the production dataset. Once the cleaning and formatting of the *sitrep* table is complete, the wildfires in the master list are moved to the production dataset and the deleted records are archived to a separate deletions file for reference (Table 16). The comparison resulted in the deletion of 527 sitreps from the first historical dataset (3.7%, 57% of these overlapping with Historical System 2) and 3,597 sitreps from the second historical dataset (3.4%).

We found a duplication issue in the *Current System* when reporting extended into the following year. For some incidents, some or all of the associated sitreps were being duplicated and assigned a new unique system identifier. We manually inspected the records to determine if it made sense to delete one of the incidents or merge the two into a single incident. This resulted in the deletion of 108 incidents (527 sitreps) and the merging of 49 overlapping incidents. The deleted records are archived in a deletions file (see output files in Table 16). Details about duplicate incident identifiers can be found in the Incident Cleaning File (ics-inc-cleanup{yyyytoyyyy}.csv) included with the source files.

### Record repair methods 2014+

There were issues with the dataset between 2015-2017 that were unresolvable at the time of our prior release. Structure and resource data was missing in 2015 and 2016 because it had been overwritten with data from 2014. In some cases, the sitreps in 2015 and 2016 are also missing important values within the reports such as INCIDENT_NAME or INCIDENT_NUMBER. There is significant data loss across all reports within these years. Additionally, the point of origin coordinates in 2017 were corrupted with random values across individual incidents.

We worked with contacts at the National Interagency Coordination Center to update the overwritten files. We patched the sitreps using values from the SIT209_HISTORY_INCIDENTS table. This allowed us to fix the static information such as *incident name*, *incident number, discovery date, cause*, and all the location description fields including the *point of origin coordinates*. The situation reports in 2015 and 2016 are missing narrative text and other content that we were unable to repair, but the fill rates for these fields are roughly equivalent with other years. We use these coordinates to fix the inconsistencies within reports in 2017.

### Merging and final cleaning

Once each of the individual datasets is refined, historical data elements are renamed to align with the corresponding columns in the current version (Table 10) and the individual *Incident Status Summary* tables are appended into a single dataset. Unused columns are dropped.

**Table 10:** Columns Renamed from Historical Systems 1 & 2.

Current Column Name	Historical System 2 Name	Historical System 1 Name
ADDTNL_COOP_ASSIST_ORG_NARR	COOP_AGENCIES	
CURR_INC_AREA_UOM	AREA	
CURR_INCIDENT_AREA	AREA_MEASUREMENT	
DISCOVERY_DATE	START_DATE	STARTDATE
DISPATCH_PRIORITY	DISPATCH_PRIORITY	DPRIORITY
EST_IM_COST_TO_DATE	COSTS_TO_DATE	ECOSTS
EXPECTED_CONTAINMENT_DATE	EXP_CONTAIN	CDATE
GACC_PRIORITY	GACC_PRIORITY	GPRIORITY
HAZARDS_MATLS_INVOLVMENT_NARR	FUELS	
INC_MGMT_ORG_ABBREV	IMT_TYPE	
INC_MGMT_ORG_DESC	IMT_TYPE_DESC	TEAMTYPE
INCIDENT_COMMANDERS_NARR	IC_NAME	TEAMNAME
INCIDENT_NAME	INCIDENT_NAME	ENAME
INCIDENT_NUMBER	INCIDENT_NUMBER	EVENT_ID
INCTYP_ABBREVIATION	INCTYP_DESC	ITYPE
INCTYP_DESC	INCTYP_DESC	INCTYP_DESC
PCT_CONTAINED_COMPLETED	P_CONTAIN	F_CONTAIN
POO_LATITUDE	LATITUDE	LATDEG + LATMIN
POO_LONGITUDE	LONGITUDE	LONGDEG + LONGMIN
POO_SHORT_LOCATION_DESC	LOCATION	LOCATE
POO_STATE	UN_USTATE	UN_USTATE
PROJ_INC_AREA_UOM	EST_FINAL_AREA	
PROJ_INCIDENT_AREA	AREA_MEASUREMENT	
PROJ_SIG_RES_DEMOB_START_DATE	DEMOBE_START	
PROJECTED_FINAL_IM_COST	EST_FINAL_COSTS	
REMARKS	REMARKS	NARRATIVE
REPORT_TO_DATE	REPORT_DATE + HOUR	REPDATE
SIGNIF_EVENTS_SUMMARY	SIG_EVENT	
**TOTAL_PERSONNEL**	TOTAL_PERSONNEL	PERSONNEL
UNIT_OR_OTHER_NARR	UN_UNITID	UN_UNITID

The script then makes a final cleaning and smoothing pass across the records, filling missing values where appropriate and smoothing data elements to make them more consistent. The specifics are described below.

### Filling missing values

Several fields in the dataset are either cumulative or the value, once known, is unlikely to change. We forward filled these fields with the previous known value to minimize gaps and to make sure that these values were propagated to the final report. This was important not just for consistency, but also because these records are used to produce the *Wildfire Incident Summary* table described below. Forward filled fields include: *Acres, Estimated Incident Management Costs to Date, Fatalities, Injuries to Date, Latitude, Longitude, Projected Final Incident Management Costs, Total Structures Damaged, Total Commercial Structures Damaged, Total Residential Structures Damaged, Total Structures Destroyed, Total Commercial Structures Destroyed*, and *Total Residential Structures Destroyed*.

### Smoothing acres and calculating new acres

Once the forward-filling of acres is complete, we perform a backwards smoothing pass. If an estimate of *Acres* is reduced on a subsequent report, we reduce the number of acres on previous reports given that a fire never truly gets smaller; this is to address overestimations at the time of reporting. After making the necessary adjustments, we use the values to populate the *New Acres* field, which is then used to calculate the daily fire spread rate (*Wildfire FSR*) (see Wildfire Incident Summary section below).

### Smoothing cost estimates

The consistency of the cost attributes is critical for analysis, even if the estimates are subject to bias and real-time information is limited. The reported values are still useful for comparing estimated costs across incidents in the research dataset. Both the *Estimated Incident Management Costs to Date* and the *Projected Final Incident Management Cost* fields were sparsely populated, with the *Estimated Incident Management Costs to Date* populated only 35% of the time and the *Projected Final Incident Management Cost* only 2% of the time. This field was also particularly prone to data entry error and variations in notation, particularly for estimates in the millions or billions of dollars. When the records were sorted by incident and report date, it was easy to identify instances where someone either left off a digit or added too many for that particular day. Also, as cost increased, sometimes notation changed to simplify data entry (e.g., 1,200,000 becomes 1.2 for $1.2 million dollars). After forward filling the values, we started the cleaning process by manually inspecting all instances where the final reported values were an order of magnitude smaller than the maximum value entered across the reports. We designated the final value based on comparison of trends across the existing reports. We were conservative, only correcting obvious errors. These updates are individually updated in the *cost adjustments* function of the merge script. Once corrections were made to the final reported values, we performed two smoothing passes. We first worked backward from the final cost, adjusting any estimates that were more than 10x larger than the current value by reducing it until it was within the 10x limit. We then worked forward, adjusting any values that were at least 9x smaller than the previous estimate until they fell within the 9x limit. When both these passes were complete, if there was no value for the *Projected Final Incident Management Cost*, we defaulted it to the *Estimated Incident Management Costs to Date* on the final report.

### Creating the wildfire incident summary record

The cleaned and merged *Incident Status Summary* table is used to create the *Wildfire Incident Summary* table. This table extracts key elements from the individual sitreps to describe the fire and support high-level analysis across wildfire events. This information includes the cause, discovery date information, final acres, final estimated costs, injuries, fatalities, if evacuations recorded at any point during the fire, and the point of origin (latitude/longitude) for the fire. The summary also includes metrics for threats to life and property, structures damaged/destroyed, and firefighting response. In the cases of threats and response, the daily/regular Incident Status Summary (sitrep) values cannot be summed to get a true accounting of things like total personnel assigned. Instead, we sum these values across sitreps to provide indices representing the level of threat or response across the duration of the incident. We also identify peak volumes and corresponding days across the fire including peak personnel, peak aerial response, and peak fire spread. Finally, we calculate what we call the *Cessation Date* when the fire grew to within 95% of its final size. This metric is valuable because the containment date may be quite conservative, with incident management teams hesitant to declare a fire contained until there is very limited risk of further growth.

### Linking to additional wildland fire datasets

The co-authorship of this paper made linking with the Fire Program Analysis Fire-Occurrence Database (FPA FOD^42,43^) a logical extension of the ICS-209-PLUS dataset. The FPA FOD is a compilation of final fire reports from the federal, state, and local fire services. The wildfire records include a final determination for location (i.e., point of origin), cause, discovery and containment dates, and fire size. It also provides connectivity to MTBS^44,45^. The MTBS products, in turn, provide satellite-based wildland fire perimeter and burn severity data.

The *Incident ID* is used to join *Wildfire Incident Summary* records with records in the FPA FOD Extract file. This file is an excel spreadsheet published as part of the dataset using the naming convention FOD_JOIN_{mmddyyyy}.xlsx. Matching records between the two datasets was an iterative process, with a focus on fires >100 acres having a clearly defined point of origin. At time of publication, 86% of all incidents in the *Wildfire Incident Summary* table (regardless of size) link to at least one FPA FOD record. As we continue to clean and refine the dataset we will publish incremental updates to this file^35^.

In the latest release we use a combination of the FPA FOD Extract file and the Wildfire Complex Configurations file. The columns used to link to the FPA FOD are described below (Table 11) and the columns added to the *Wildfire Incident Summary* are described in (Table 12).

**Table 11:** Columns used from the FPA FOD Extract File - Column Descriptions.

Column Name	Description
ICS_209_PLUS_JOIN_ID	Concatenated Incident ID Linking an FPA FOD Record to an ICS-209-PLUS Incident or Complex
ICS_209_PLUS_COMPLEX_JOIN_ID	Concatenated Incident ID Linking an FPA FOD Record to an ICS-209-PLUS Complex
CONT_DOY	Containment Date Counted from First Day of Calendar Year
DISCOVERY_DOY	Discovery Date Counted from First Day of Calendar Year
FIRE_NAME	Recorded Name for Wildfire
FIRE_SIZE	Fire Size in Acres
FOD_ID	Unique Identifier in the FPA FOD Database
LATITUDE	Latitude Coordinate for the Reported Point of Origin
LONGITUDE	Longitude Coordinate for the Reported Point of Origin
MTBS_ID	MTBS ID Associated with the Fire or Complex
MTBS_FIRE_NAME	MTBS Fire Name Associated with the Fire or Complex
NWCG_GENERAL_CAUSE	NWCG General Cause Description: *Arson/incendiarism, Debris and open burning, Equipment and vehicle use, Firearms and explosives use, Fireworks, Missing data/not specified/undetermined, Misuse of fire by a minor, Natural, Other causes, Power generation/transmission/distribution, Railroad operations and maintenance, Recreation and ceremony, Smoking*

**Table 12:** New Fields from FPA FOD Added to the Wildfire Incident Summary Table.

Column Name	Description
FOD_CAUSE	List of FOD Causes Associated with the Incident
FOD_CAUSE_NUM	The Number of FOD Causes
FOD_COMPLEX_NAME	Fire Complex Name
FOD_CONTAINMENT_DOY	Day of Year for Fire Containment or Minimum Value if Multiple Fires
FOD_COORD_LIST	List of FOD Coordinates Related to the Incident
FOD_COORD_NUM	Number of FOD Coordinates
FOD_DISCOVERY_DOY	Day of Year for Discovery date or Maximum Value if Multiple Fires
FOD_FIRE_LIST	List of FOD Fires Related to the incident as key/value pairs (Replaces FOD_LIST)
FOD_FIRE_SIZE	Final Size of the Fire or Sum of Acres if Multiple Fires (Replaces FOD_FINAL_ACRES)
FOD_FIRE_NUM	Number of FPA FOD Records Associated with Incident (Replaces FOD_NUM_FIRES)
LRGST_FOD_COORDS	Coordinates Related to Largest FOD Wildfire
LRGST_FOD_LATITUDE	FOD Latitude for the Point of Origin in Decimal Degrees (Replaces FOD_LATITUDE)
LRGST_FOD_LONGITUDE	FOD Longitude for Point of Origin in Decimal Degrees (Replaces FOD_LONGITUDE)
LRGST_FOD_ID	Fire-Occurrence Database ID or Largest Fire if Multiples (Replaces FOD_ID)
MTBS_FIRE_LIST	List of MTBS Fires Associated with the Incident
MTBS_FIRE_NUM	Number of MTBS Fires Associated with the Incident

### FPA FOD and MTBS fields

The majority of incidents in the ICS-209-PLUS dataset matched with a single record in the FPA FOD (84%) but a small percentage (2%) were fire complexes associated with multiple records in the FPA FOD. To balance the potential for multiple FPA FOD identifiers per incident with the more general case, we developed the following solution: The FPA FOD values for the largest fire are joined to the ICS-209 incident-level record if there is a many-to-one relationship (i.e., multiple FPA FOD fire reports linking to the same ICS-209-PLUS Incident ID [i.e., complexes or other sitrep groupings]. The *FOD_CAUSE* field takes all unique values for cause. It is typically one but will contain a list of values if there are multiple causes across a multi-fire incident. The discovery date is the earliest value in related FPA FOD records, and the containment day is the latest. The *FOD_FINAL_ACRES* is the sum of all reported acres for joined FPA FOD fires. The FOD_FIRE_NUM field is the number of fires linked to the ICS-209-PLUS incident and *FOD_FIRE_LIST* stores detailed information about each fire related to the incident. Table 12 below describes each of the FPA FOD and MTBS fields in Wildfire Incident Summary Table.

The FOD_FIRE_LIST is in JSON format. This provides both a human-readable and machine-parsable summary at the incident level. For example, the 1999 Arizona Jump Complex has three records in the fire-occurrence database and so the *FOD Fire List* contains three associated entries:

[{**“ID”**: 215365, **“MTBS_ID”**: “AZ3662411371319990528(JUMPSPRING)”, **“COORDS”**: (36.5928,-113.7352), **“CAUSE”**: “Lightning”, **“SIZE”**: 16816.0, **“DISC”**: 148, **“CONT”**: 154.0},

{**“ID”**: 215366, **“COORDS”**: (36.6216, -113.7172), **“CAUSE”**: “Lightning”, **“SIZE”**: 7.0, **“DISC”**: 148, **“CONT”**: 154.0},

{**“ID”**: 215369, **“COORDS”**: (36.96, -113.8158), **“CAUSE”**: “Lightning”, **“SIZE”**: 69.0, **“DISC”**: 149, **“CONT”**: 150.0}]

### Creating the wildfire complex associations table

The *Wildfire Complex Associations table* spans the full timeframe for the current release and includes links to the FPA FOD and MTBS. The raw *Complex Associations* table was not added to the ICS-209 archive until 2010. Consequently, this important relationship is missing in early records. Information about wildfire complexes and the associated member wildfires are derived from a combination of historic linking files including the *Short Master List*, the *FOD Extract File*, and the annual *Complex Associations tables*. This custom version of the C*omplex Associations* table accomplishes multiple objectives. It provides continuity across the entire historic record and connectivity to MTBS and the FPA FOD at the complex level. This linkage is important for incidents that don’t map directly to any records in the FPA FOD. For example, the 2020 Arroyo fire exists in the ICS-209-PLUS dataset as Incident ID 2020_11875360_ARROYO but there is no corresponding record in the FOD Extract file because the acreage for the Arroyo Fire is accounted for under the SCU Lightning Complex. The record in the *Wildfire Complex Associations table* allows us to define the Arroyo Fire as a member of the SCU Lightning Complex in the ICS-209-PLUS dataset and link it to the FPA FOD through this relationship. Additionally, the *Wildfire Complex Associations table* is more concise, complete, and easier to decipher than records in the annual *Complex Associations tables*. In the current system there are more than two hundred thousand records in the latter. Multiple table joins are needed to understand how member fires and complexes relate one another and most of these complexes have no corresponding sitreps in the sitrep table. By including the FOD Extract File we increase the number of fire complexes defined by 31% and member fires by 69%.

The FPA FOD Extract file defines relationships between individual fires and fire complexes in the FPA FOD. The Wildfire Complex Configurations file defines relationships between fire complexes and member fires within the ICS-209-PLUS dataset. The Wildfire Complex Configurations file is published with source the files using the naming convention cpx-assocs{yyyytoyyyy}.csv. This file is copied verbatim as the foundation for the *Wildfire Complex Associations* table which is then linked to the FPA FOD and MTBS through the FOD_FIRE_LIST and MTBS_FIRELIST attributes respectively (see Table 13).

**Table 13:** Wildfire Complex Associations Table.

Column Name	Description
COMPLEX_INC_IDENTIFIER	Unique System Identifier for the Complex (2014 + only)
COMPLEX_INCIDENT_ID	Incident Id Value for the Fire Complex or Member Fire
FIRE_YEAR	Final Year for the Fire Complex
FIRE_NAME	Fire Name for the Fire Complex or Member Fire
FOD_FIRE_LIST	List of fires associated with Fire Complex in the FOD Database
FODJ_INCIDENT_ID	Incident Identifier Linking the Fire Complex or Member Fire to FOD Join File
ICS_COMPLEX_NAME	Complex Name
INCIDENT_NAME	Incident Name for Each Incident Incorporated into Fire Complex (1999–2013)
INCIDENT_NUMBER	Incident Number for Each Incident Incorporated into Fire Complex (1999–2013)
ICS_COMPLEX_NAME	Final Name Given to the ICS Complex
MEMBER_INC_IDENTIFIER	Unique Incident Identifier for the Member Incident (2014 + only)
MEMBER_INCIDENT_ID	Incident Id Value for the Member Incident (2014 + only)
MTBS_FIRE_LIST	List of Fires Linked to MTBS for this Fire Complex and/or Member Fire

### Linking the wildfire incident summary record to FIRED

Wildfire events are compiled from FIRED (Fire Events Delineation)^46,47^, a novel database of global wildfire perimeters derived from the Moderate Resolution Imaging Spectrometer (MODIS) burned area product (MCD64A1) (2001-2020)^48,49^. These satellite-derived wildfire events provide additional information not captured in MTBS, including smaller wildfires below MTBS’s defined burn area thresholds (≤1000 acres in the Western US. and ≤500 acres in the Eastern US); estimates of the daily fire growth patterns such as the simple fire spread rate (acres/day); maximum single-day fire growth (acres); and the point of estimated ignition location (or the coordinate of the first identified burned pixel). Additionally, FIRED wildfire burn footprints can distinguish geographic areas *within* the burn footprint that may not have burned, a distinction that MTBS footprints do not always make. Linking *Wildfire Incident Summary Records* (hereafter “wildfire incidents”) to corresponding FIRED events involves a multi-step approach that processes four distinct subsets of wildfire incidents. At each step of the process, previously joined wildfire incidents are removed to avoid duplication.

First, wildfire incidents that are not associated with a complex (single incidents) are matched to their associated MTBS footprint using the linkage from the FPA FOD. Using these footprints, corresponding FIRED events are identified using a spatial overlay where the largest spatially overlapping polygon in the same fire year is retained. Matches were discarded if they exceeded an ignition date difference greater than 25 days and exceeded a difference of burned area greater than either 50,000 absolute burned acres or a 50% difference in final burned acres, relative to the FPA FOD’s reported metrics. For each matched incident-FIRED event pair, the distance between wildfire incident Point of Origin (POO) and FIRED event centroid is calculated to inform the following steps.

Second, wildfire complex incidents are processed separately, using the *Wildfire Complex Associations table* (see section above) to retain the final complex incident report and discard member incident reports. Because satellite-derived events often map complexes as a single perimeter, we consider the final complex incident report to be the most accurate in describing overall area burned. This set of wildfire complex incidents are converted to spatial points using their POO coordinates. From each POO, a 25 km buffer is created. This buffer distance was selected based on the average and standard deviation of distance between wildfire incident POO and FIRED event centroids calculated from the first set of wildfire incidents described above. For each year, all FIRED events intersecting a given wildfire incident buffer are identified and filtered to retain events that match the spatial and temporal thresholds described above. This method often produces multiple corresponding FIRED events for each wildfire incident. In these instances, we retain the event which equals the minimum difference in area burned. To ensure high confidence in these buffer-based spatial joins, we then apply a more restrictive spatial threshold of 5,000 absolute acres or 35% difference in burned acres, removing potentially erroneous joins.

Third, unmatched incidents which do not have a link to the FPA FOD and which are not associated with a fire complex are processed using the same buffer-based methods and thresholds described above. After completing this process, we use a K-nearest neighbour approach and match all remaining wildfire incidents to their 50 spatially nearest neighbors that occurred in the same year and that met our established spatial and temporal thresholds. This subset of incidents includes those in which the POO coordinates may be further from a corresponding FIRED event than the buffer distance used in previous steps.

The process described above results in 14,796 incident-FIRED event pairs across the Continental United States (CONUS) and Alaska, representing 43.8% of total incidents (1999-2020). This important subset captures 81.4% of burned area, 91.4% of residential structures destroyed, and 86.3% of estimated suppression expenditures. This procedure provides burned area footprints for thousands of incidents unaccounted for by MTBS due to the dataset’s acreage thresholds for inclusion. Through the FIRED linkage, we join 7,263 additional wildfire footprints to ICS records, which provides critical spatial details for the spatiotemporal linkage (details below).

We validated the linked incidents by predicting the burned area in acres reported by the *Wildfire Incident Summary Records* using the satellite-derived burned area in FIRED events and a linear regression model (R^2^ = 0.95). This strong goodness of fit demonstrates a high confidence of correspondence between the two datasets. The code used to generate the ICS-209 to FIRED join is available as a public repository^40^. The following fields were added to the *Wildfire Incident Summary Table* (Table 14).

**Table 14:** Fields from FIRED added to the Wildfire Incident Summary Table.

Column Name	Description
FIRED_ID	Unique Identifier Linking the Incident Report to its Corresponding FIRED Event
FIRED_ACRES	Burned Area in Acres Mapped by FIRED
FIRED_SIMPLE_FSR	Simple Fire Spread Rate (Acres/Day) Measured from FIRED
FIRED_MAX_GROWTH	Maximum Single-day Fire Growth (Acres)
FIRED_IG_LAT	Latitude of the Estimated Ignition Location
FIRED_IG_LON	Longitude of the Estimated Ignition Location

### Assigning wildfire incident ids to spatial and temporal units

To facilitate analyses of wildfire impacts in conjunction with concurrent social and environmental phenomena, we link wildfire records from the ICS-209-PLUS to the relevant spatial units and temporal periods in which each incident occurred (ICS-209-PLUS spatiotemporal linkage). Each observation is uniquely identified by its GEOID (spatial unit identifier), INCIDENT_ID, quarter, and year (Table 15). The spatiotemporal linkage is available for three geographies (counties, census tracts, and census block groups) and assigns 97.9% of ICS-209-PLUS wildfire incidents to corresponding spatial and temporal units.

**Table 15:** ICS-209-PLUS Spatiotemporal Linkage Variables.

Column Name	
GEOID	Spatial Unit in which the Incident Occured (County, Tract, or Census Block Group) Taken from Year 2020 Boundaries
STATE_ABBREV	State Abbreviation Corresponding to the GEOID (e.g., CA)
COUNTY_NAME	County Name Corresponding to the GEOID (e.g., Jefferson) Taken from Year 2020 boundaries
INCIDENT_ID	The ICS-209-PLUS Incident ID
QUARTER	Quarter in which the Incident Occured
YEAR	Year in which the Incident Occured
SPATIAL_DATA_ORIGIN	Indicates whether a Wildfire Incident’s Corresponding Spatial Unit was Determined by Point of Origin (POO), MTBS Perimeter, FIRED Perimeter, or Both MTBS and FIRED Perimeter

To produce the linkage, we exclude prescribed fires (Incident Type = RX) and incidents created to track multiple fires contained during the initial attack phase. We then assign each incident to corresponding spatial units based on each incident’s available MTBS ID, FIRED ID, and/or point of origin. We use fire perimeters from the MTBS Burned Areas Boundaries Dataset released on April 28, 2022, and FIRED fire perimeter data^46,47^. If neither MTBS ID nor FIRED ID is available for an incident, it is assigned only to the spatial unit in which its reported point of origin resides. Boundaries for these spatial units are all taken from the year 2020 and do not account for changes in spatial units over time^50^. The variable SPATIAL_DATA_ORIGIN (Table 15) indicates how each GEOID was assigned (by MTBS footprint, FIRED footprint, both, or only POO). Via the INCIDENT_ID field, users can then join the spatiotemporal linkage to the full suite of incident variables contained in the ICS-209-PLUS incident tables.

The ICS-209-PLUS spatiotemporal linkage allows researchers to aggregate wildfire impacts across different spatial and temporal scales. This ability offers several advantages over one of the few national datasets that documents wildfire-related losses, the Spatial Hazard Events and Losses Database for the United States (SHELDUS)^51^. First, the spatiotemporal linkage provides finer spatial resolution than SHELDUS’s county scale, including estimates for counties as well as for census tracts and census block groups. Second, the spatiotemporal linkage to the ICS-209-PLUS reports direct damage metrics (e.g., counts of structures destroyed) instead of monetary losses (e.g., dollar value of structures destroyed). Measures of direct damage avoid the property bias embedded in monetary loss metrics, in which physical damage to a higher-value property is weighted more heavily than an equal amount of physical damage to a lower-value property. Direct damage metrics further avoid temporal bias that can emerge in longitudinal hazard data, in which monetary losses are not adjusted for inflation over time, and thus appear higher in more recent reporting^52^.

**Fig. 2:**
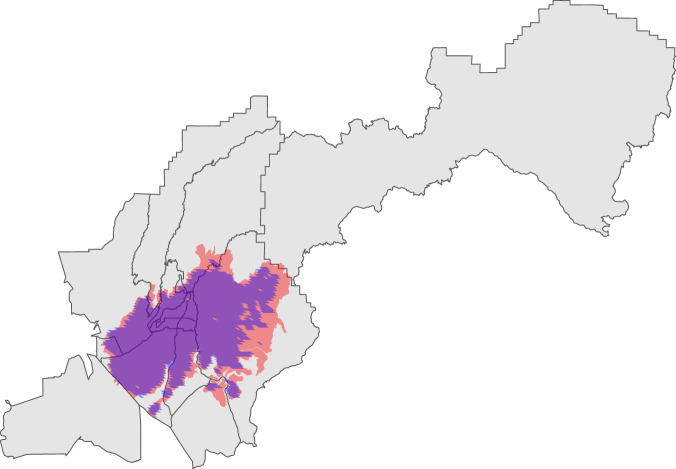
Perimeters of the 2018 California Camp Fire taken from MTBS (shown in red) and FIRED (shown in purple), and displayed with corresponding census tracts from the spatiotemporal linkage (shown in grey).

When aggregating the spatiotemporally linked ICS-209-PLUS data, it is important for users to note that wildfire perimeters may cross multiple spatial boundaries, particularly among larger fires and for smaller spatial units (see Fig. 2 as an example). Similarly, the duration of a wildfire may cross temporal boundaries, burning over multiple quarters or years. A simple join between the spatiotemporal linkage and ICS-209-PLUS incident tables will not allocate variable values across spatial units or over temporal periods, and instead will yield fire-wide totals for each spatial and temporal unit in which a given incident occurs. For instance, if a wildfire burned across two adjacent census tracts and destroyed five buildings, the spatiotemporal linkage will associate each census tract with five destroyed buildings. Similarly, in the case of a wildfire that burned over the course of two quarters and destroyed five buildings, the spatiotemporal linkage will associate each quarter with five destroyed buildings. This approach means that caution should be used when summarizing aggregate values across spatial or temporal units, which, in some cases, can result in double-counting. We estimate that 8.3% of wildfire incidents occur in more than one county, 14.4% occur in more than one census tract, and 17.2% occur in more than one census block group. Additionally, we estimate that 6.9% of wildfire incidents occur in more than one quarter. Researchers need to make careful decisions about how to account for this characteristic of the data when using the spatiotemporal linkage.

## Data Records

The ICS-209-PLUS dataset spans 22 years from 1999 to 2020 and contains 187,160 *Incident Status Summary* reports for 35,170 thousand all-hazard incidents. The dominant hazard in the dataset is wildland fire (98.4%) with the remaining 1.6% spread across other hazards. The number of incidents is lower overall prior to the 2005 mandate but contains roughly the same distribution of fire and non-fire incidents as in subsequent years. Given the dominance of wildfire in the dataset, we created three tables specifically for wildland fire analysis: a wildfire-specific *Incident Status Summary* table with just the wildfire sitreps, a *Wildfire Incident Summary* table with key values for each fire, and an *ICS-209-PLUS Complex Associations* table. Table 16 summarizes each of the tables in the ICS-209-PLUS dataset and the number of records contained in each. The data can be found online at figshare^35^.

**Table 16:** Tables in Production Dataset.

Dataset Table Name	Description	# Records
ics209-plus_sitreps_1999to2020	All-hazards Dataset	187,115
ics209-plus-wf_sitreps_1999to2020	All Wildfire and Prescribed Burn Sitreps Daily Incident Status Summary Records	182,826
ics209-plus-wf_incidents_1999to2020	Incident-level Summary for All Wildfire Incidents	34,622
ics209-plus-wf_complex_associations_1999to2020	Complex Associations Summary Record for ICS-209-PLUS Wildfire Complexes	4,764
ics209-plus-wf_incidents_by_county_1999to2020	Spatiotemporal Linkage by County, Quarter/Year Resolution	40,688
ics209-plus-wf_incidents_by_tract_1999to2020	Spatiotemporal Linkage by Census Tract, Quarter/Year Resolution	45,245
ics209-plus-wf_incidents_by_cbg_1999to2020	Spatiotemporal Linkage by Census Block Group, Quarter/Year Resolution	48,370
ics209_sitreps_deleted_hist1_1999to2002	Deleted Records from HIST1	579
ics209_sitreps_deleted_hist2_2001to2013	Deleted Records from HIST2	3,575
ics209_sitreps_deleted_curr_2014to2020	Deleted Records from Current *Subject to Change	572

## Technical Validation

***It is important to note that the ICS-209-PLUS dataset represents a small but important subset of wildfires (1***–***2%). The patterns presented in this technical validation are only for large or otherwise significant incidents and not meant to be interpreted as holding for wildfires in general****.*

### Wildfire distribution

**Fig. 3:**
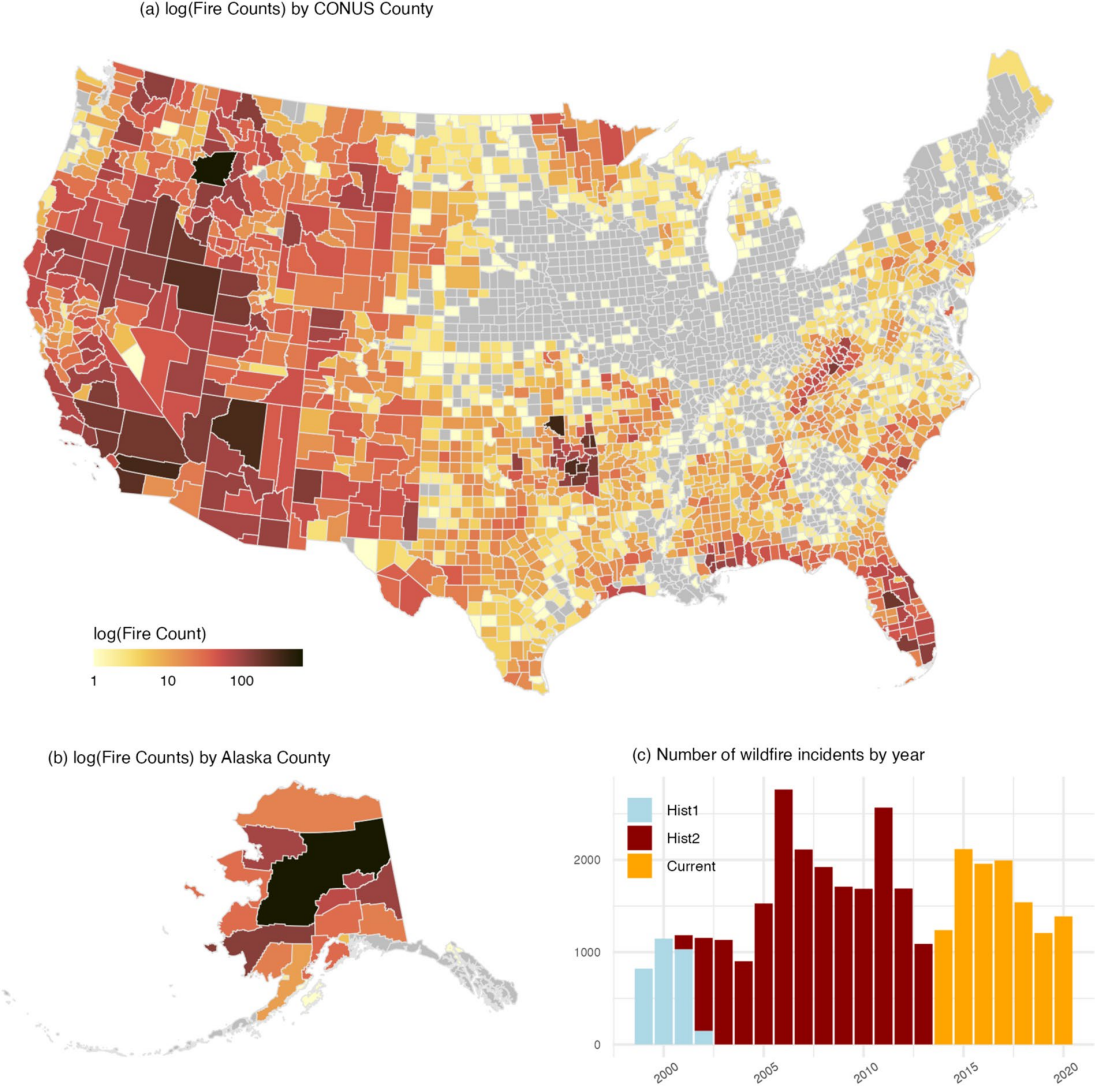
Log-scaled (**a**) number of incidents by conterminous US (CONUS) county, (**b**) number of incidents by Alaska county, and (**c**) number of incidents by year from each of the historical releases of the ICS-209 reports. Grey color denotes counties with no associated incidents during the time-period. Incidents were assigned to a county based on the Point of Origin (POO) coordinates in the ICS-209-PLUS dataset.

Wildfires requiring ICS-209 reporting are most numerous in the interior of Alaska and continental US with hotspots in parts of California, the Northern Rockies, Northern Forests, and parts of the South-eastern U.S (Fig. 3). Fire reporting is notably limited in areas of the Central Midwest and North-eastern US (Fig. 3), and there were relatively few fires reported from in Hawaii and Puerto Rico (not shown).

### Spatial distribution across key variables

**Fig. 4:**
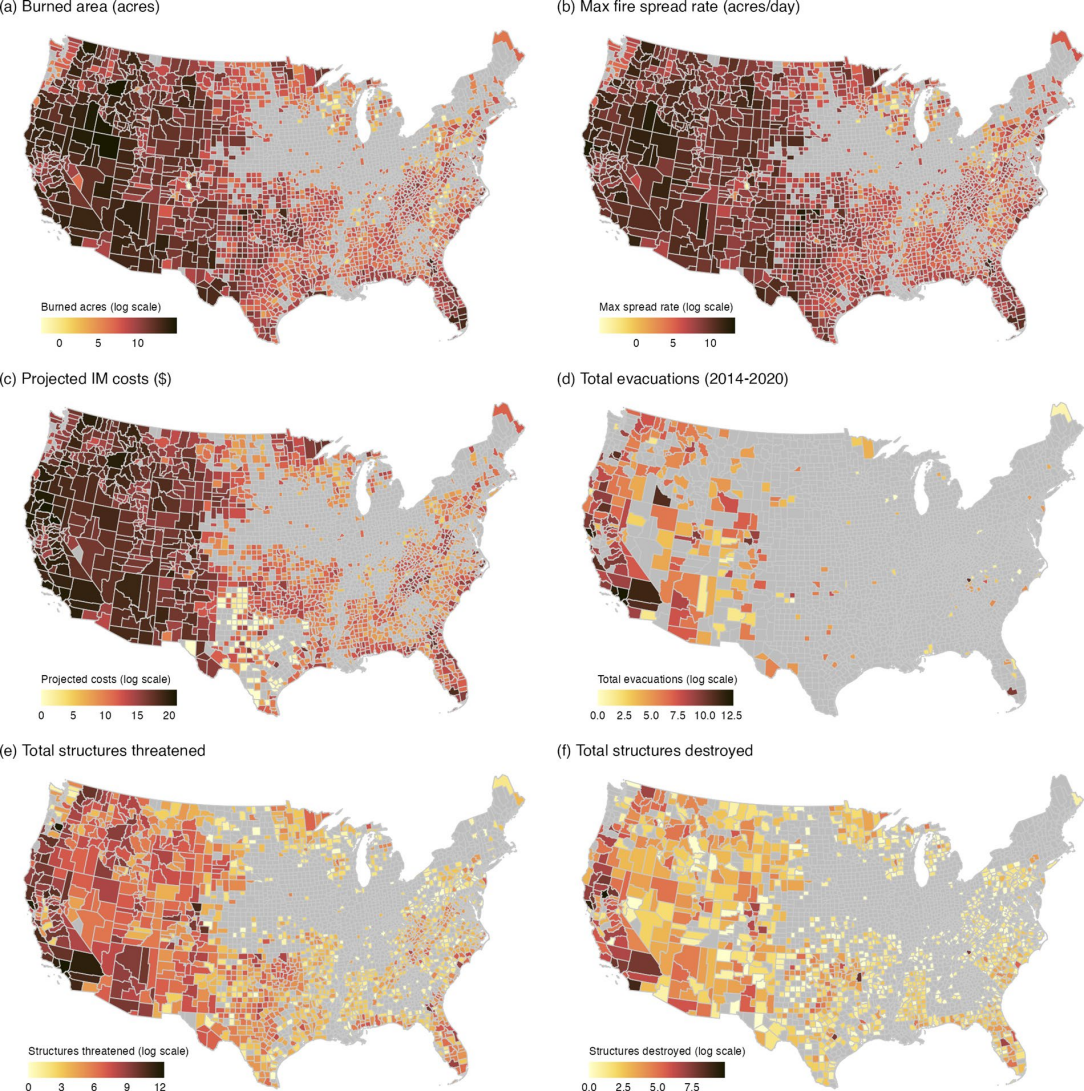
Spatial distribution of key variables for CONUS counties. Log-scaled (**a**) burned area (acres), (**b**) average maximum fire spread rate (acres/day), (**c**) projected incident management costs ($), (**d)** total assigned personnel × report, (**e**) total number of threatened structures, and (**f**) total destroyed structures. Spatial distributions for Alaska counties not shown here but can be calculated from the data product. Incidents were assigned to a county based on the Point of Origin (POO) coordinates in the ICS-209-PLUS.

We examined the spatial distribution across six variables within the dataset: burned area, maximum fire spread rate, estimated incident management costs, total assigned personnel (described below), maximum structures threatened, and the number of structures destroyed (Fig. 4). The fastest-spreading fires were in the northern Great Basin area, along the border of Nevada and Idaho, where landscapes are dominated by fast-burning sagebrush and cheatgrass fuels. Additionally, the West experienced larger, faster fires requiring more resources and resulting in higher suppression costs. Suppression costs are an order of magnitude lower on average in the East versus the West, but societally impactful fires are not limited to the West (Fig. 4e,f). Smaller fires in the East threaten and destroy large numbers of homes. The allocation of firefighting personnel is greatest in California and the Pacific Northwest, with California committing the most resources regionally. This allocation of resources may help to lessen the number of homes damaged or destroyed, particularly across densely populated fire-prone landscapes. Where it may not reduce destruction is in the hottest and driest portion of Southern California. Wildfires requiring ICS-209 reporting have a presence in all but parts of the midwest and northeast. In the Appalachian Mountain range, the influence of human ignitions has led, in recent years, to large areas burned and associated ICS-209 reporting. More research is needed to better understand the factors driving these apparent patterns.

Summary statistics were generated across the national Geographic Area Coordination Center (GACC) boundaries (https://gacc.nifc.gov/) for the six key metrics plus fire count and mean fire size, and total personnel (Table 17). Alaska had the largest reported average maximum fire spread rate and average fire size, despite having second lowest fire count. The numbers reported for *personnel* have been summed across status reports. This roughly approximates the number of personnel shifts worked and threat accumulated over days rather than physical headcount or structures respectively. In CONUS, the Northwest region experienced the fastest fires, with an average maximum fire spread rate of 3,566 acres/day. The average fire size in the southern GACC (southeastern US) was 869 acres, or roughly 85% smaller overall. Though wildfires were substantially smaller in the southern GACC, it experienced the most total fire incidents (13,423) and third most destroyed structures (16,741). The state of California (Northern and Southern Ops) account for 45% of the total reported suppression costs, 49% of total personnel assigned, 53% of all structures threatened, 62% of all structures destroyed, yet only 15% of the total burned area across all GACCs.

**Table 17:** Summary statistics of key variables across national Geographic Area Coordination Centers (GACCs).

GACC	Fire Count	Total Burned Area (acres)	Mean Fire Size	Mean Max Fire Spread Rate	Total Suppression Costs ($)	Total Personnel × Reports	Total Structures Threatened	Total Structures Destroyed
Alaska	1,342	22,928,955	17,086	4,056	$986,438,648	569,150	35,257	645
Eastern	2,809	1,346,420	479	276	$118,161,872	153,041	16,824	1,850
Great Basin	3,957	25,397,448	6,418	3,302	$3,120,718,114	2,613,938	155,207	2,802
Northern Ops	1,184	11,173,964	9,437	3,492	$8,414,979,219	6,893,345	557,856	48,596
Northern Rockies	2,361	9,428,221	3,993	1,634	$2,557,877,794	2,110,144	67,639	2,058
Northwest	1,958	16,035,146	8,190	3,566	$6,018,233,404	4,190,469	458,862	7,485
Rocky Mountain	1,939	6,975,426	3,597	1,954	$1,903,336,134	1,135,050	207,898	5,582
Southern	13,423	11,667,096	869	539	$793,172,866	980,387	193,674	16,741
Southern Ops	2,116	8,114,045	3,835	1,448	$6,048,717,809	6,338,065	919,492	19,021
Southwest	3,130	18,849,679	6,022	2,871	$2,188,210,342	1,981,622	158,843	3,805
Totals	34,219	131,916,401	5,993	2,314	$32,149,846,203	26,965,212	2,771,551	108,585

### National interagency fire center (nifc) comparison

We compared the ICS-209-PLUS dataset with annual fire statistics provided by the National Interagency Fire Center (www.nifc.gov) across the same time period. The ICS-209-PLUS dataset captured approximately 2% (range 0.6% to 3.5% annually) of the population of wildfires, accounting for approximately 80% of the acres burned (range 53% to 98% of acres burned annually) and 79% of the suppression costs (range 51% to 140% of costs annually). These numbers are roughly in line with expectations, but the annual variability indicates that there are still significant outliers in the values that are skewing the results.

### Case study

#### The 2017 chetco bar fire

The following summary is derived from the Government Accountability Office (GAO) report to congressional requesters (2020)^53^. The Chetco Bar fire was ignited by lightning in the Kalmiopsis Wilderness of Oregon. It was reported by an airline pilot on July 12th, 2017. The fire was burning primarily in the burn scar of the 2002 Biscuit Fire in steep, difficult-to-access terrain. A small crew of firefighters were deployed by helicopter on July 12th and again on the morning of July 13th. Helicopters dropped water on the fire, but by the afternoon of July 13th firefighting resources were pulled out citing safety concerns and a low probability of containment. By July 20th the fire had grown to just over 300 acres forcing the closure of USFS roads and trails. In August, increasing temperatures and fire growth led to further closures and restrictions in wilderness areas. On August 17th, strong, hot, dry winds known as a Brookings or Chetco effect^54^ began to drive fire growth and the fire crossed the Chetco River. On August 18th the fire grew dramatically in size and evacuations began near the town of Brookings, Oregon. Over the next few days evacuations continued to expand and by August 20th over 3,000 residents had been affected, six homes destroyed, and the fire grew to over 90,000 acres. By mid-to-late September cooler temperatures and increased moisture helped moderate the fire growth. It was declared fully contained on November 2, 2017. At its peak, over 800 firefighting resources were assigned to the fire, with over $69 million in suppression costs^55^.

**Fig. 5:**
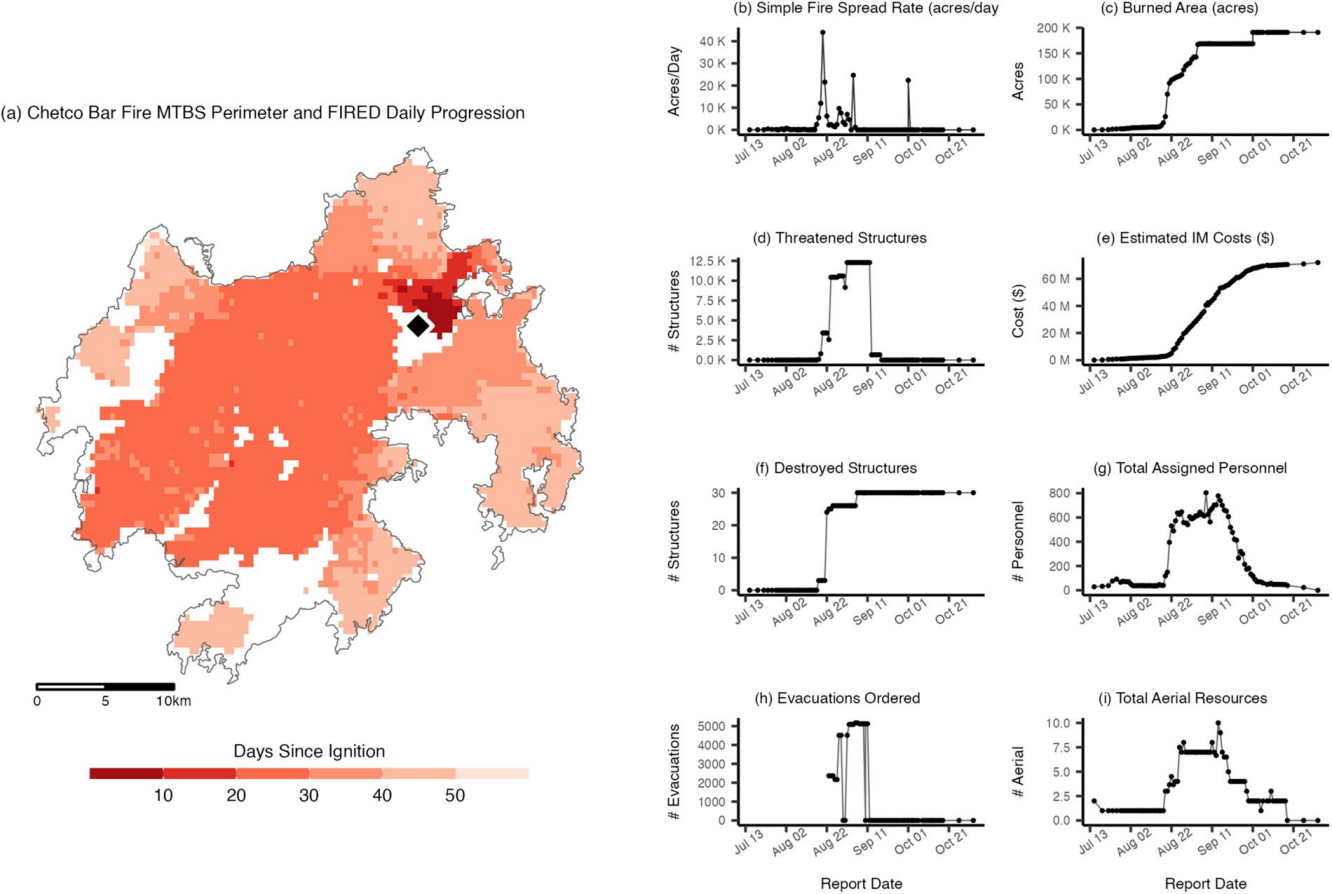
2017 Chetco Bar Fire case study. (**a**) Chetco Bar wildfire perimeter from Monitoring Trends in Burn Severity (MTBS) denoted in black outline, ICS-209-PLUS Point of Origin (POO) denoted in black diamond, and the satellite-derived wildfire progression map clustered into 10-day burn periods (since ignition) from the MCD64A1 and linked through FIRED events. (**b**–**i**) selected key variables from the ICS-209-PLUS situation reports which outline resources and values at risk throughout the incident duration. The Chetco Bar Fire reports maximum growth 39 days from the discovery date coinciding with increases in resource allocation and values at risk.

There are 99 situation reports for the Chetco Bar fire spanning July 15, shortly after discovery, through final containment on November 2. Looking across key metrics on the 209 reports, one can see this fire narrative play out (Fig. 5). There is a close correlation across the peaks of key variables. There is a slight lag in the reporting of burned area, which may reflect delays in finalising estimates, particularly during the most volatile phases of large and fast-spreading fires. The onset of Brookings-effect winds is observable in the Max FSR (5b), driving a steep incline in fire growth (5c). These spikes correspond with structural threat (5d) and home destruction (5e). The estimated incident management costs rise steeply during the active growth phase of the fire and level out at $46 million dollars. These estimates are likely to be conservative, particularly on large, fast-moving events with values based on what is known at the moment rather than a clear accounting of final cost. It may take months for the final numbers to be tallied on large-scale responses, and therefore are expected to differ in situation reports and final fire-reporting systems. The allocation of firefighting personnel ramps up steeply to just over 5,000 on September 1st, and then begins to taper off later in September. The number of structures threatened rises quickly, with the first threat reported as 25 structures at 6 pm on August 19th. This value jumps to 2,505 at 7am the following morning with a total of seven structures destroyed including two residences. Structures threatened rose again on August 23rd to 4,503 at 6am and 5,503 reported at 7:30 pm, coinciding with new evacuation advisories.

The values logged for both total personnel and structures threatened may represent the most knowable information in-the-moment. The tracking of personnel happens in real-time during a shift, and concrete estimates of homes and areas threatened by a wildfire are integral to incident management decisions. It is also likely that there is a lag between reported resource needs and the deployment of new firefighting personnel. We hypothesise that the first report of structures threatened may serve as an accurate indicator of the onset of social disruption and that the growth and levelling out of personnel indicates that the fire is in its most acute and socially disruptive phase with the onset of demobilisation of resources indicating the easing of the threat posed by a fire. In this case, the first structural threat is recorded at 7 pm on August 17th and jumps to 3,418 on August 20th. This figure reaches its peak at over 12,000 structures on September 1st, where it remains for twelve days. The number of personnel steadily increases through mid-September when resources begin to steadily drop as conditions ease.

The perimeter and progression map (5a) visually depicts the rapid growth of the fire. The black diamond inside the perimeter at the top right is the Point of Origin latitude/longitude from the *Wildfire Incident Summary Report*. The final perimeter outline is from the MTBS dataset^44,45^ and the fire progression is constructed using the MODIS burned area data^48,49^ linked through the FIRED ID^46,47^. Because the MODIS data records the last burn detection for each pixel, the maximum growth is recorded on day five of the fire, which is earlier than the formal reporting of this same acreage on the situation report. This highlights two important points. Knowing that there is a likely delay in the reporting of acres burned on the sitreps, we may be able to make adjustments to these values as part of any analysis. Additionally, given the strengths and weaknesses of the values in ICS-209 reporting and the inherent biases, users should consider applications of the ICS-209-PLUS dataset in conjunction with other wildfire data products, such as those from remote sensing platforms, when intending to relate fire-spread events to socioeconomic impacts and firefighting response.

## Usage Notes

Wildfire activity has increased in the US over the past several decades^5–8^, and the ICS-209-PLUS dataset affords valuable, science-grade, situation-reporting information regarding the development and consequences of large or otherwise significant wildfire events. This dataset provides a unique perspective that augments other important data sources, from after-action agency fire reports (FPA-FOD^42,43^) to satellite-based detections of active fire and burned area (i.e., from Landsat, MODIS, VIIRS^46,48,56^).

Although the incidents in this dataset represent only 2% of all wildfires, they account for approximately 80% of suppression costs. The daily/regular situation reports capture the best in-the-moment information on fire growth and behavior, environmental conditions such as weather and terrain, firefighting response, and the built and natural assets at risk. As exposure of highly valued resources and assets to wildfire increases, data on this important population of fires provides opportunities to understand relationships between a changing fire environment, incident response levels, and potential social and ecological impacts.

This first revision (second edition) of the data product meets several key objectives. It aligns the underlying data model across the three versions of the system, such that the data product now spans 1999 to 2020. Substantial cleaning efforts and filling of missing values allow for more robust analyses across the full domain. Additionally, the enhancements in the open-source code used to create this dataset is open source and can easily be extended to process and add subsequent years to the product. More work is needed to streamline the data processing and expedite publication phases. Instead of processing annually archived data, there is potential to build capability to avail research-ready situation reporting information in near-real time for analyses and integration with other in-the-moment data, including from surface-weather stations and remote-sensing platforms.

One of the longstanding barriers to using the ICS-209 electronic archive has been the data quality and lack of alignment across application versions. As in most human-generated, observational datasets, much of the data has been entered free form and is difficult to standardize for research purposes after-the-fact. Our goal was to strike a balance between manual inspection and cleaning and programmatic data compilation. We focused manual efforts on high-value fields like those related to fire location and outcomes like size and cost, for which we felt cleaning would provide the highest initial return. We automated the standardisation of values across the reporting systems and auto-filled empty values wherever it made sense. Given their intended in-the-moment uses, the data will likely continue to require post-hoc refinement for research purposes. Means for improved quality control and near-real-time compilation could greatly improve the scientific value of the data, as research interests and analytical needs grow within the fire service.

Our hope is that making this dataset available will lead to cross-sector and cross-discipline work resulting in greater understanding of our nation’s wildfire activity and response patterns, and associated impacts. Understanding the causes and consequences of wildfire is a complex task requiring expertise across disciplines and potentially benefiting those in the forefront of fire management, climate science, environmental hazards research, policy making, planning and development. The ICS-209-PLUS dataset provides an important level of detail that can be used in parallel with other sources of information, filling in gaps and providing a completer and more nuanced picture of the relationship between characteristics of wildfire, incident response, and the causes and consequences of threatening wildfires across the nation. There is potential to address a critical information need as we work to understand trends and address the impacts and consequences of fire in an evolving physical and social landscape. This dataset has a great future research benefit, particularly if current limitations are addressed effectively through community-wide efforts to keep improving on this rich dataset in an open science framework.

## Data Availability

The source code used to create the ICS-209-PLUS dataset, the spatiotemporal linkage, and the ICS-FIRED linked database are publicly accessible on GitHub^39–41^.
